# Imputation of missing values for cochlear implant candidate audiometric data and potential applications

**DOI:** 10.1371/journal.pone.0281337

**Published:** 2023-02-06

**Authors:** Cole Pavelchek, Andrew P. Michelson, Amit Walia, Amanda Ortmann, Jacques Herzog, Craig A. Buchman, Matthew A. Shew

**Affiliations:** 1 Department of Otolaryngology Head and Neck Surgery, Washington University School of Medicine, St. Louis, Missouri, United States of America; 2 Institute for Informatics, Washington University School of Medicine, St. Louis, Missouri, United States of America; 3 Department of Pulmonary Critical Care, Washington University School of Medicine, St. Louis, Missouri, United States of America; Sejong University, REPUBLIC OF KOREA

## Abstract

**Objective:**

Assess the real-world performance of popular imputation algorithms on cochlear implant (CI) candidate audiometric data.

**Methods:**

7,451 audiograms from patients undergoing CI candidacy evaluation were pooled from 32 institutions with complete case analysis yielding 1,304 audiograms. Imputation model performance was assessed with nested cross-validation on randomly generated sparse datasets with various amounts of missing data, distributions of sparsity, and dataset sizes. A threshold for safe imputation was defined as root mean square error (RMSE) <10dB. Models included univariate imputation, interpolation, multiple imputation by chained equations (MICE), k-nearest neighbors, gradient boosted trees, and neural networks.

**Results:**

Greater quantities of missing data were associated with worse performance. Sparsity in audiometric data is not uniformly distributed, as inter-octave frequencies are less commonly tested. With 3–8 missing features per instance, a real-world sparsity distribution was associated with significantly better performance compared to other sparsity distributions (Δ RMSE 0.3 dB– 5.8 dB, non-overlapping 99% confidence intervals). With a real-world sparsity distribution, models were able to safely impute up to 6 missing datapoints in an 11-frequency audiogram. MICE consistently outperformed other models across all metrics and sparsity distributions (p < 0.01, Wilcoxon rank sum test). With sparsity capped at 6 missing features per audiogram but otherwise equivalent to the raw dataset, MICE imputed with RMSE of 7.83 dB [95% CI 7.81–7.86]. Imputing up to 6 missing features captures 99.3% of the audiograms in our dataset, allowing for a 5.7-fold increase in dataset size (1,304 to 7,399 audiograms) as compared with complete case analysis.

**Conclusion:**

Precision medicine will inevitably play an integral role in the future of hearing healthcare. These methods are data dependent, and rigorously validated imputation models are a key tool for maximizing datasets. Using the largest CI audiogram dataset to-date, we demonstrate that in a real-world scenario MICE can safely impute missing data for the vast majority (>99%) of audiograms with RMSE well below a clinically significant threshold of 10dB. Evaluation across a range of dataset sizes and sparsity distributions suggests a high degree of generalizability to future applications.

## Introduction

Cochlear implantation (CI) is considered one of modern medicine’s highest achievements, capable of restoring hearing in patients with severe-to-profound hearing loss. As of December 2019, there have been over 736,000 registered devices implanted worldwide; in the United States, approximately 118,100 adults and 65,000 children have been implanted [1]. However, despite this success, big data research in CI patient care is essentially non-existent. Published studies typically range from single digits to 100–200 patients due to non-standardized protocols, missing data, and lack of collaboration [2,3]. This has major implications for outcomes research, population monitoring, and identification of potential CI candidates.

Data-centric statistical approaches are providing new opportunities to operationalize big data to improve patient care. In the field of CI research, machine learning models may offer novel insights into CI speech performance prognostication. However, missing data must first be appropriately dealt with [4]. Few studies directly address the development and validation of imputation methods, with potentially drastic implications for prediction model performance [4–6]. Missing data can bias results and lead to inefficient analyses though loss of precision and power [7,8]. Common methods for handling missing data include complete case analysis, missing indicators, and univariate imputation. While simple, these methods can introduce significant bias [5,6,9,10]. More sophisticated multivariate models use subjects’ other known characteristics to increase imputation accuracy [5].

In addition to model selection, imputation accuracy depends on structural characteristics of data, such as feature intercorrelation, dataset size, amount of missing data, and distribution of sparsity [8]. However, patterns of missing audiometric data in patients undergoing CI evaluation have yet to be thoroughly characterized. Prior work on imputing missing audiometric data is also limited. Charih *et al* compared linear interpolation to k-nearest neighbors (KNN) for prediction of 3000Hz and 6000Hz with no difference in mean absolute error (MAE) found [11]. Reported MAE ranged from 5.38 dB to 7.36 dB depending on the model and frequency output. However, all atypical audiograms were algorithmically excluded from analysis using a gaussian mixture model, potentially inflating performance. Pitathawatchai *et al* compared models for prediction of certain frequencies in 206 pediatric audiograms using specific input frequencies [12]. They found that machine learning models such as neural networks and KNN were in aggregate (MAE 5–8 dB) superior to linear interpolation (MAE 6.25–10 dB). However, the small sample size and homogenous pediatric population limits generalizability.

Notably, all prior studies have assessed prediction of specific frequency outputs using specific frequencies as input. No study has attempted to simulate real-world patterns of missing data for imputation model assessment. This presents a significant barrier to implementation, as an understanding of real-world model performance is a critical prerequisite for use in clinical research. CI candidates also represent a potentially more challenging subset of patients, as varying degrees and etiologies of hearing loss can lead to more variance in audiometric thresholds as compared with the general population. While of particular interest, to date no study has looked at imputing missing data in this specific population. Finally, the field of machine learning, particularly in the context of medical data, suffers from a lack of standardization of methods to rigorously validate models. If imputation is to be employed to leverage larger datasets for CI research, proper validation of imputation techniques is critical to ensure results are generalizable, clinically relevant, and statistically sound.

The contributions of the study are as follows:

1. Proposal of a model for imputation model validation on randomly generated sparse datasets with structural sparsity equivalent to real-world data. Publication of code to facilitate validation of real-world imputation model performance on novel datasets.
2. Assessment of the independent effects of sparsity distribution, amount of missing data, and dataset size on imputation model performance.
3. Demonstration that, for this dataset, up to 6 missing frequencies per 11-frequency audiogram can be safely imputed, allowing for a 5.7-fold increase in sample size.
4. Demonstration that, depending on dataset size and sparsity, either interpolation or MICE is optimal for audiometric imputation; complex, black-box machine learning models are unnecessary.
5. We make available the largest CI audiometric dataset to-date, which may be used for outcomes modeling and to bolster imputation accuracy for other audiometric datasets.

## Methods

### Study population and source of data

Approval was obtained by author’s local Institutional Review Board with waiver of consent (IRB#202108009) at Washington University in St. Louis. Audiograms were acquired from HERMES (Auditory Implant Initiative, Wichita Falls TX, www.aii-hermes.org) and the Washington University School of Medicine in St. Louis (WUSM) CI data registry. HERMES is a national, prospective, web-based CI database including 32 private practice and academic institutions that collects de-identified data including age, sex, ethnicity, etiology and duration of hearing loss, speech perception testing, and audiograms [13]. The WUSM CI data registry captures similar information but is limited to one institution. All routine audiometric testing was done by licensed doctors of Audiology and performed in soundproof booths. Audiogram analysis was limited to post-lingual deafened and native English-speaking adults (≥18 years of age) undergoing cochlear implant evaluation. Eleven frequencies were included as features: 125 Hz, 250 Hz, 500 Hz, 750 Hz, 1000 Hz, 1500 Hz, 2000 Hz, 3000 Hz, 4000 Hz, 6000 Hz, and 8000 Hz.

**Fig 1:**
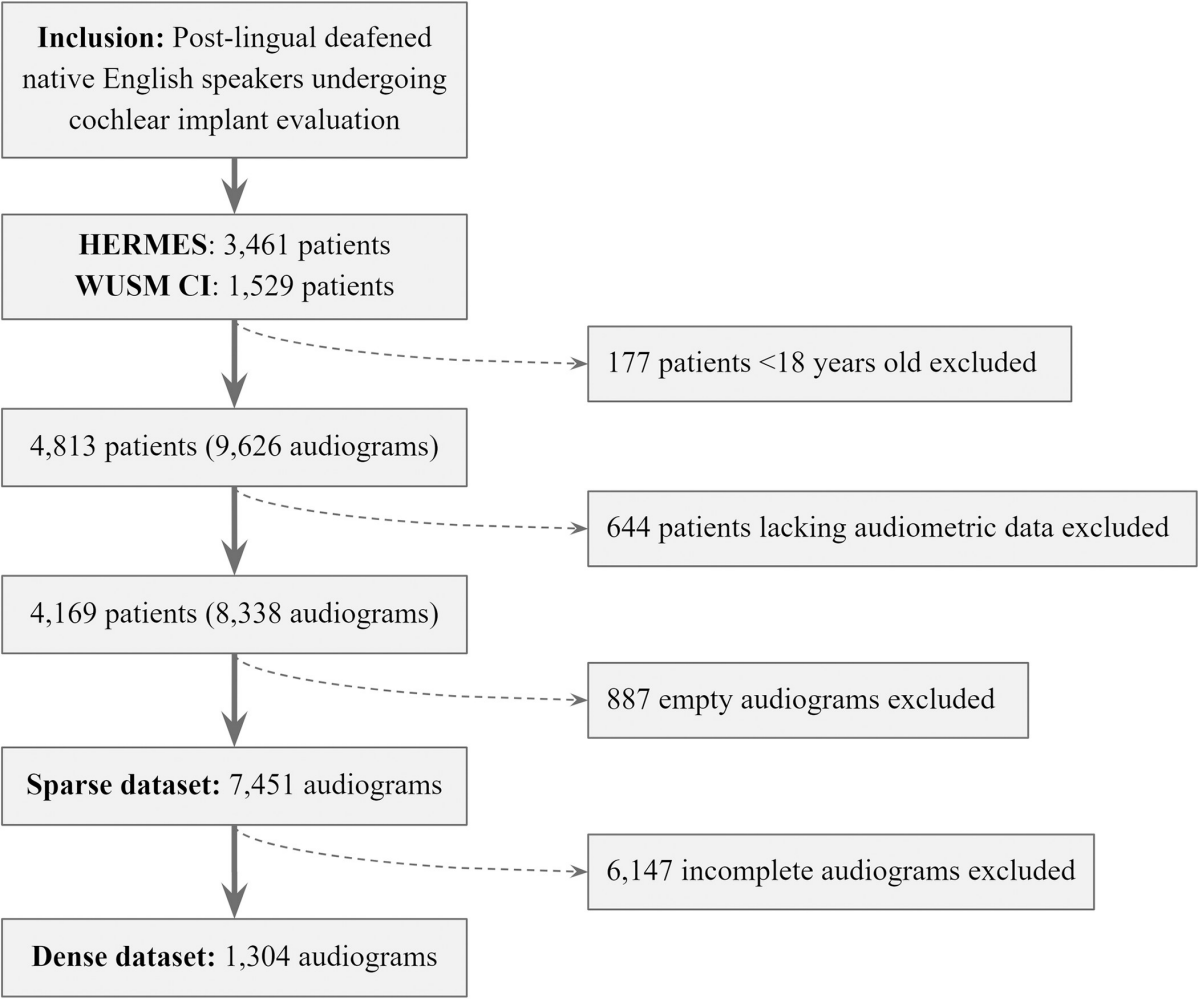
Dataset creation. Flow chart for inclusion and exclusion of subjects and audiograms. Data drawn from the Washington University School of Medicine in St. Louis Cochlear Implant database (WUSM CI) and the HIPPA-secure, Encrypted, Research, Management, and Evaluation Solution database (HERMES).

Invalid values (e.g., text or extreme numbers) were removed. Audiogram values were clipped to a range of 0 dB to 120 dB to account for inter-site variation in the reporting of extreme values. “No response” thresholds were imputed as 120 dB, the maximum value recorded by most audiometers. Removal of blank instances yielded a sparse “parent” dataset of 7,451 audiograms, used to calculate the distribution and degree of sparsity in real-world CI audiometric data. Complete case analysis yielded a dense dataset of 1,304 audiograms for model assessment (Fig 1). The sparse parent dataset with associated demographic data is available in supplementary files.

### Model assessment

**Fig 2:**
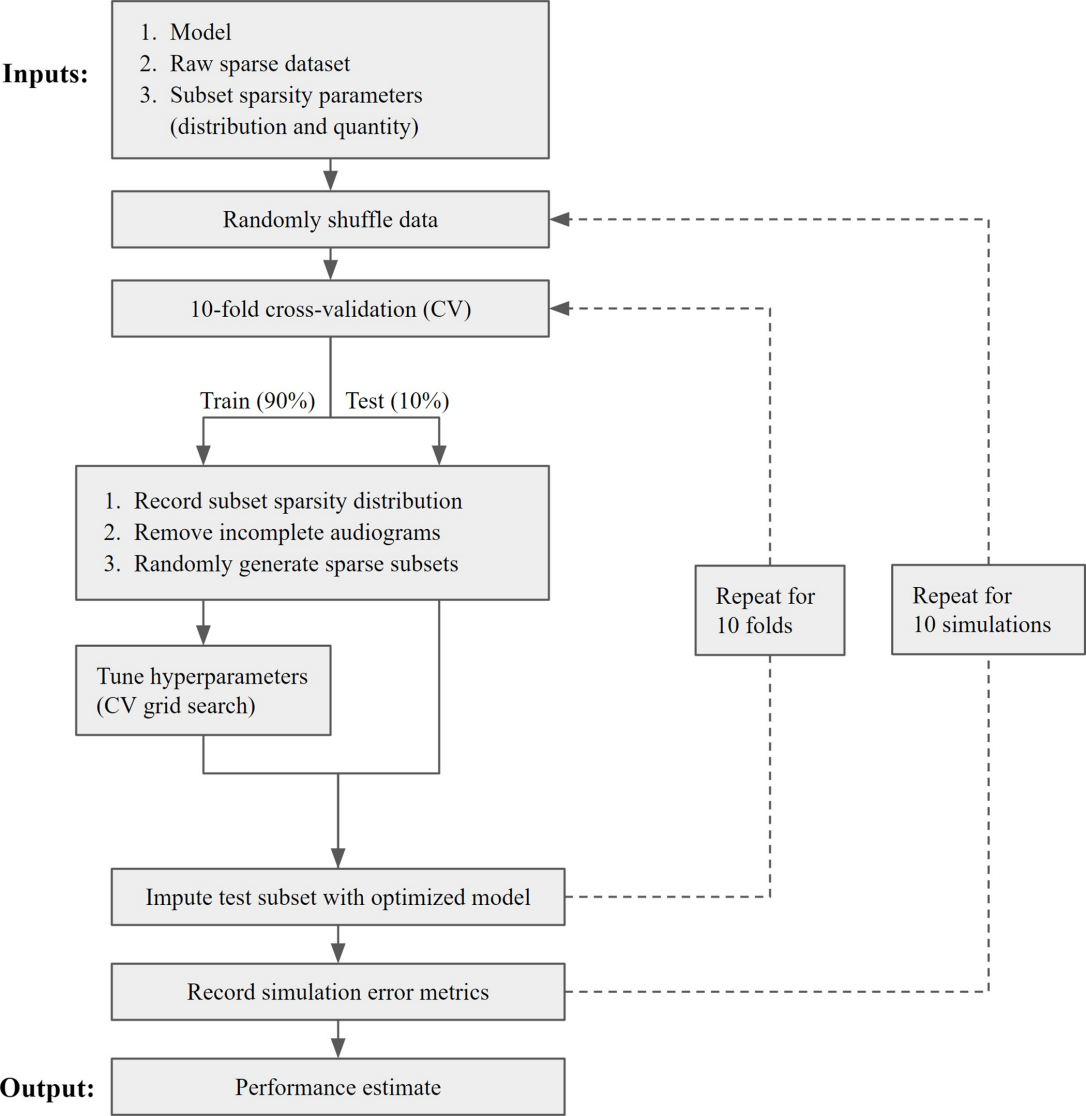
Performance assessment pipeline. Diagram of steps for assessment of model performance with repeated simulations of nested cross-validation. Output consists of model performance mean and confidence intervals averaged across all 10 simulations.

Models were assessed with 10 simulations of nested 10-fold by 10-fold cross-validation (Fig 2). 10-fold cross validation has been shown to represent a good trade-off between bias, variance, and computational cost [14]. Instances were randomly shuffled prior to each simulation such that fold partitions varied from run to run, as reporting the average and significance of repeated runs of k-fold cross-validation has been shown to significantly decrease variance of results [15]. Additionally, repeating runs helps account for the stochasticity of randomly generated sparse datasets. Nested cross-validation allows for model assessment across the entire dataset while avoiding data leakage from hyperparameter optimization, leading to more accurate performance estimates [16].

**Fig 3:**
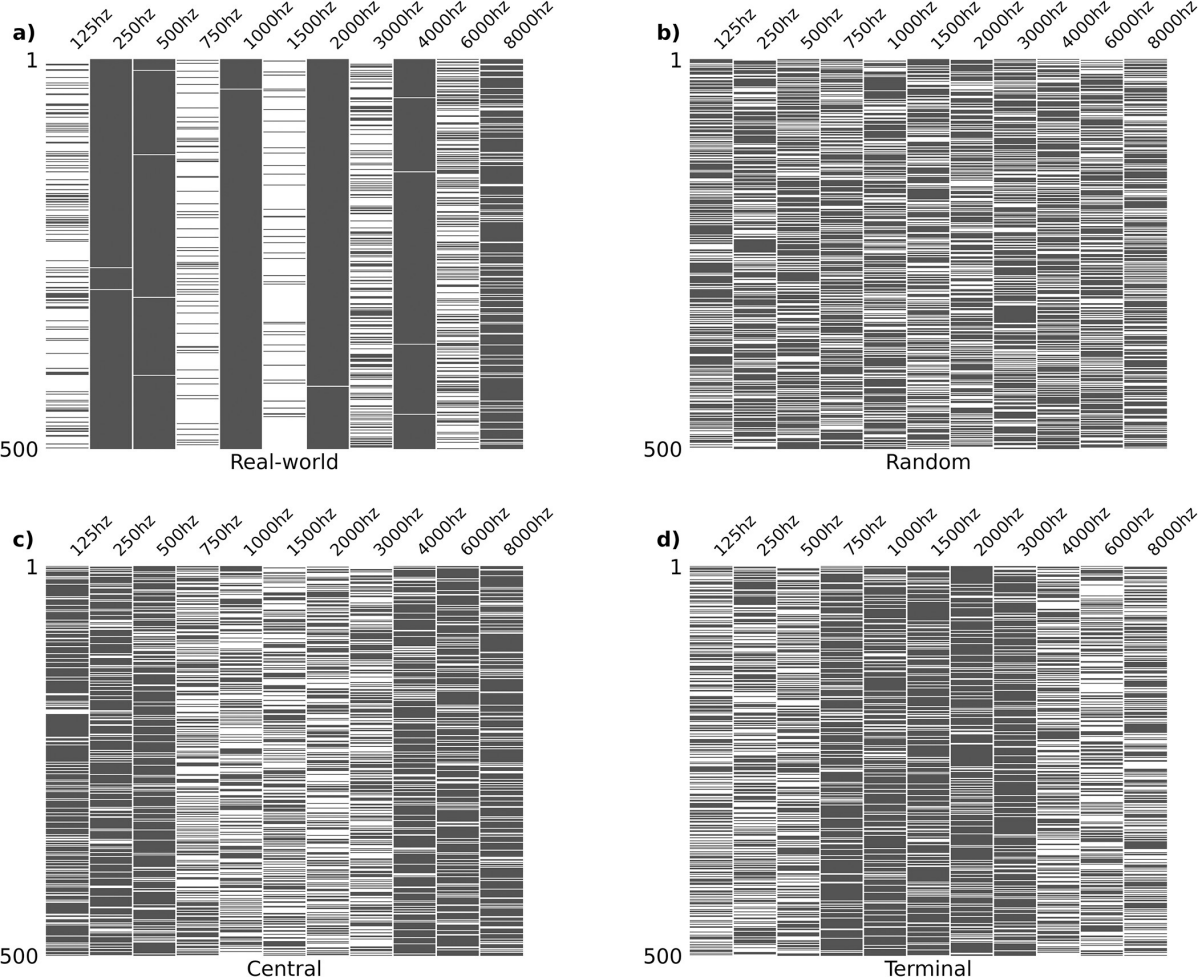
Visualization of sparsity distribution. All tested sparsity distributions applied to sample dataset of 500 audiograms. Quantity of sparsity fixed at 3 missing features per instance. Rows represent audiograms, columns represent features. Black bars indicate present data, white bars indicate missing data. (a) Real-world distribution sets weighted likelihood for feature removal equivalent to the corresponding parent subset. (b) Random distribution sets equivalent likelihood of removal for all features. (c) Terminal distribution is weighted 3-fold towards removing the terminal 6 frequencies (125 Hz, 250 Hz, 500 Hz, 4000 Hz, 6000 Hz, 8000 Hz). (d) Central distribution is weighted 3-fold towards removing the central 5 frequencies (750 Hz, 1000 Hz, 1500 Hz, 2000 Hz, 3000 Hz).

For each fold, the parent dataset (n = 7,451) was divided into training (90%) and test (10%) subsets, with test subsets sampled without replacement across folds. Sparsity distribution was calculated independently for each subset; incomplete instances were subsequently removed to create dense subsets. This was done following the split into train and test subsets to avoid potential data leakage. Sparse datasets with known underlying values were created from dense subsets by randomly deleting values from each instance. When randomly generating sparse datasets, two axes of sparsity were independently controlled for: **quantity** (how many features are missing per instance) and **distribution** (which features are relatively more likely to be missing). Four sparsity distributions were tested (Fig 3). Tunable hyperparameters were optimized on the randomly generated sparse train subset with 10-fold cross-validation (S1 Table). The optimized model was used to predict imputations for the sparse test subset. Simulation performance metrics were measured across all 10 test subsets in aggregate. The mean and confidence interval of model performance across all simulations was reported as output.

### Metrics

Coefficient of determination (R^2^ score) describes the proportion of variance explained by the model (Eq 1). R^2^ score is useful for comparing performance across dependent variables with varying degrees of noise. Mean absolute error (MAE) is the average absolute magnitude of residual error (Eq 2). While directly interpretable, MAE is robust to outliers, potentially masking critically high individual errors. Root mean squared error (RMSE) is the square root of the sum of squared residual errors (Eq 3). The squared error term more heavily penalizes higher individual errors, giving a more conservative estimate of performance.


$$R^{2}\;score=1-\frac{\sum_{i=1}^{n}{\left(y_{i}-f_{i}\right)}^{2}}{\sum_{i=1}^{n}{\left(y_{i}-\bar{y}\right)}^{2}}$$ (1)

$$MAE=\frac{\sum_{i=1}^{n}\left|y_{i}-f_{i}\right|}{n}$$ (2)

$$RMSE=\sqrt{\frac{\sum_{i=1}^{n}{\left(y_{i}-f_{i}\right)}^{2}}{n}}$$ (3)

*f*_*i*_ = *i*th *predicted value*

*y*_*i*_ = *i*th *true value*

$\bar{y}$ = *data mean*

*n* = *number of datapoints*

### Error threshold

We defined a clinically meaningful change in an audiometric threshold as ≥10dB, based on two factors. First, the established intra-session test-retest reliability of routine audiometry is +/- 5dB [17,18]. Second, the American Speech-Language-Hearing Association (ASHA) defines a clinically significant hearing change for ototoxicity monitoring as 10dB across two frequencies or 20dB in one frequency [19]. The test-retest reliability of +/- 5dB introduces intrinsic noise, representing a theoretical lower bound for model MAE. Assuming the +/- 5dB of noise holds true for this dataset, model performance significantly below this threshold would indicate overfitting or data leakage. The 10dB clinically significant threshold is used as an upper bound to determine the amount of missing data that can be safely imputed while avoiding clinically significant bias. RMSE is used to define the upper bound as it is more conservative.

### Imputation algorithms

Six models were evaluated: univariate imputation (UI), interpolation (INT), k-nearest neighbors (KNN), multiple imputation by chained equations (MICE), neural networks (NN), and gradient boosted trees (XGB). INT was implemented with the SciPy scientific computing library version 1.7 [20]. UI, KNN, MICE, and NN models were implemented with the Scikit-learn machine learning library version 1.0.2 [21]. XGB was implemented with the XGBoost Python API version 1.6.1 [22]. All testing was performed using the Python programming language version 3.9.12 (Python Software Foundation).

### Univariate imputation

UI is a single-round imputation method that imputes values for a feature using only that same feature dimension. UI is a common alternative to complete case analysis, representing an important benchmark for the performance of more complex models. The UI hyperparameter search space included the imputation strategy: feature mean, median, and mode (S1 Table).

### Interpolation

INT imputes missing data using non-missing data from that same instance only (S1 Fig). Interpolants defined between adjacent non-missing datapoints are used to impute interposed missing datapoints. Missing datapoints outside the range of non-missing datapoints are imputed by extrapolating the nearest interpolant. Linear interpolation defines interpolants as first-degree linear equations between two known coordinates (x_0,_ y_0_) and (x_2,_ y_2_). For x_1_ in the interval (x_0_, x_2_), y_1_ is defined by Eq 4. Cubic spline and piecewise cubic hermite interpolating polynomial (PCHIP) generate piecewise cubic polynomials as interpolants (Eq 5). Cubic spline interpolants have continuous first and second derivatives. PCHIP interpolants are continuous in the first derivative only, avoiding overshooting and preserving monotonicity. The INT hyperparameter search space included the interpolation method: linear, PCHIP, and cubic spline (S1 Table).


$$y_{1}=\frac{y_{2}-y_{0}}{x_{2}-x_{0}}*(x_{1}-x_{0})+y_{0}$$ (4)

$$S(x)=\left\{ \begin{array}{l} a_{0}x^{3}+b_{0}x^{2}+c_{0}x+d_{0},\;x\leq x_{1}\\ a_{1}x^{3}+b_{1}x^{2}+c_{1}x+d_{1},\;x_{1}<x\leq x_{2}\\ \;\ldots \\ a_{i-1}x^{3}+b_{i-1}x^{2}+c_{i-1}x+d_{i-1},\;x_{i-1}<x \end{array}\right.$$ (5)

*i* = *number of nonmissing features*

*a*_*i*_, *b*_*i*_, *c*_*i*_, *d*_*i*_ = *parameters for* i*th cubic polynomial*

*x*_*i*_ = *i*th *nonmissing datapoint*

### K-nearest neighbors

KNN is a single-round imputation method which imputes missing values using the k instances most similar to the instance being imputed. For a sparse instance x_s_, distance from an instance x_i_ is calculated as the average distance in Euclidean space for all shared non-missing features. The nearest neighbors are defined as the *k* instances with the lowest averaged distances from x_s_. Missing features in x_s_ are imputed using the unweighted or distance-weighted means of the k-nearest neighbors (Eq 6). The unweighted model assigns an identical weight $w=\frac{1}{k}$ to each k-nearest neighbor, such the relative importance of each nearest neighbor is equivalent. The weighted model assigns a weight to the *i*th nearest neighbor inversely proportional to the distance between x_i_ and x_s_ (Eqs 7 and 8). The KNN hyperparameter search space included the number of neighbors and the weighting function (S1 Table).


$$f_{xs}=\sum_{i=1}^{k}{w_{i}*f}_{xi}$$ (6)

$$w_{i}=\frac{1}{k}*\frac{1}{d(x_{s}-x_{i})}$$ (7)

$$\sum_{i=1}^{k}w_{i}=1$$ (8)

*x*_*s*_ = *Sparse instance to be imputed*

*x*_*i*_ = i*th nearest neighbor to x*_*s*_

*w*_*i*_ = *weight of* i*th instance*

*k* = *number of nearest neighbors*

*d*(*x*_*s*_, *x*_*i*_) = *average feature distance between x*_*s*_ *and x*_*i*_

### Gradient boosted trees

Decision trees are visualizable as a flowchart of yes or no decisions. While decision trees tend to overfit and are often poorly generalizable, ensembles are more robust [23]. Gradient boosted trees are a type of ensemble wherein individual decision trees are iteratively added to the model such that each new tree reduces the loss function, accounting for the error of the existing ensemble. We used the gradient boosted trees model XGBoost (XGB), described in Tianqi et al, 2016 [22]. As XGBoost outputs a single prediction, separate models were trained for each feature using the Scikit-learn multioutput regressor [24]. The XGB hyperparameter search space included the number of estimators, maximum tree depth, learning rate, and subsample proportion (S1 Table).

### Neural network

A feed-forward multilayer perceptron was modeled after the NN described in Pitathawatchai *et al* (Adam optimizer, rectified linear unit activation function), shown to be superior to the common approach for audiometric frequency prediction in pediatric audiograms [12,25]. As NNs are intolerant of missing inputs, missing values were denoted with -1. The NN hyperparameter search space included the number of hidden layers, the number of nodes in each hidden layer, and the learning rate (S1 Table).

### Multiple imputation by chained equations

Multiple imputation by chained equations is a well-validated method for missing data imputation [26]. Initially, all missing values are imputed using single imputation. Subsequently, all originally missing values are iteratively re-imputed by a series of multivariate regression models that impute values in a given feature using all other features as inputs. We tested l1 and l2 regularized linear regression models as the estimator (Eqs 9 and 10), as both are appropriate for data with high degrees of multicollinearity. Values are imputed for each feature in a round-robin fashion; imputation of every feature once represents a single round of imputation. This process iteratively repeats for *m* rounds, generating *m* imputations for each originally missing value. The final round of imputation, presumed to be the most accurate, is commonly used as model output. Alternatively, Single Center Imputation from Multiple Chained Equations (SICE) defines model output as the mean of all imputations [27]. The MICE hyperparameter search space included the estimator, number of iterations, initial imputation function, and output method (S1 Table).


$$l1\;loss=\frac{1}{n}\sum_{i=1}^{n}{(f}_{i}-{y_{i})}^{2}+\lambda \sum_{j=1}^{m}\left|\theta_{j}\right|$$ (9)

$$l2\;loss=\frac{1}{n}\sum_{i=1}^{n}{(f}_{i}-{y_{i})}^{2}+\lambda \sum_{j=1}^{m}\theta_{j}^{2}$$ (10)

*n* = *number of datapoints*

f_i_ = ith predicted value

*y*_*i*_ = *ith true value*

m = number of covariates

λ = *strength of regularization penalty*

*θ*_*j*_ = *weight of j*th *parameter*

## Results and discussion

### Demographics

**Fig 4:**
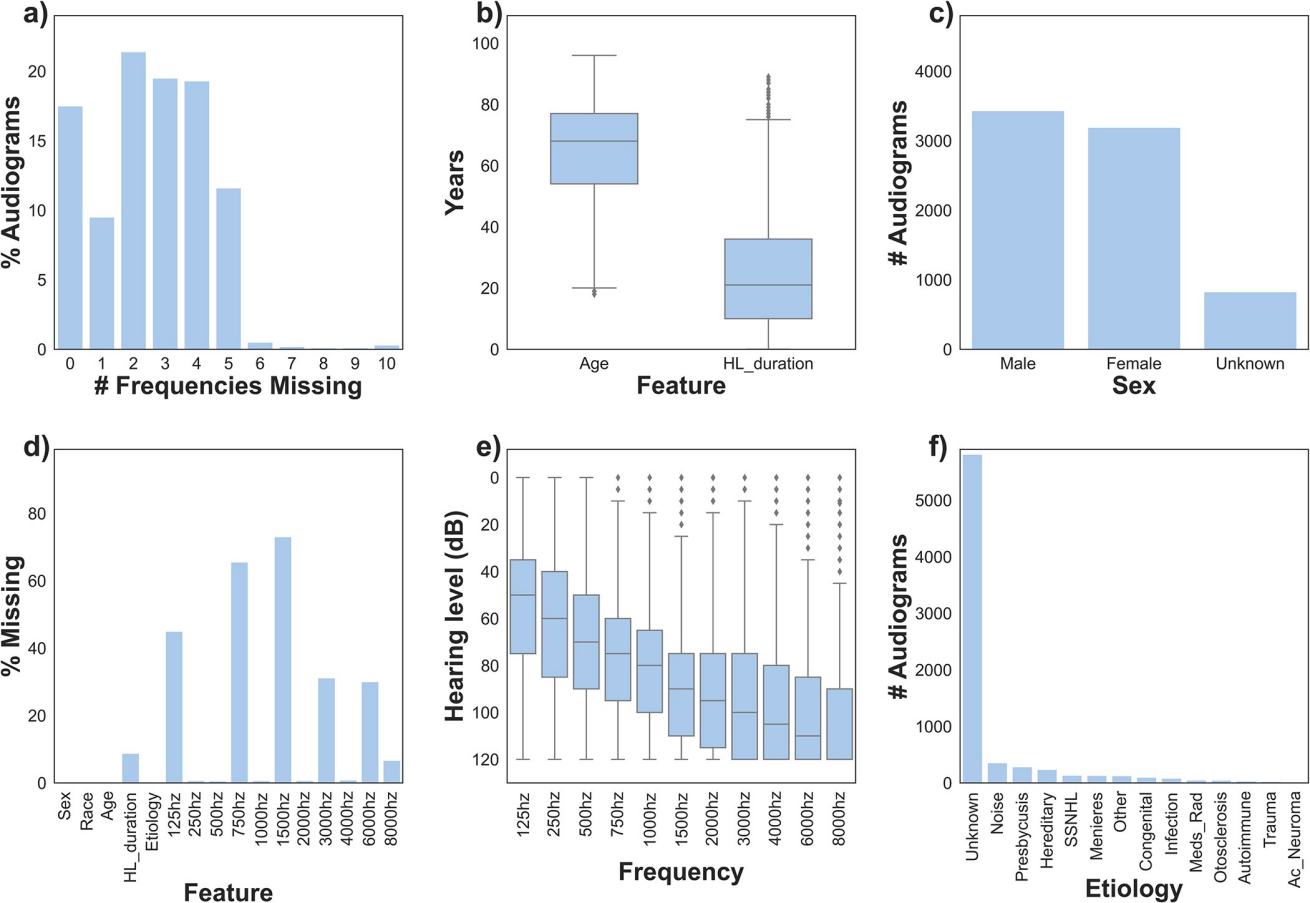
Demographics. Descriptive statistics for raw sparse audiometric dataset (n = 7,451).

Median age was 67 years (IQR 54–76 years) and median hearing loss duration was 23 years (IQR 11–37 years). Etiology was unknown in 78.0% of cases; etiology of remaining cases consisted of noise (4.8%), presbycusis (3.8%), hereditary (3.2%), sudden sensorineural hearing loss (1.8%), Meniere’s disease (1.8%), “other” (1.7%), congenital (1.4%), infection (1.1%), iatrogenic (0.7%), otosclerosis (0.7%), autoimmune (0.5%), trauma (0.3%), and acoustic neuroma (0.2%). Most audiograms were missing six or fewer frequencies (99.3%). The most commonly missing frequencies were 1500Hz (72.9%), 750Hz (65.4%), 125Hz (45.0%), 3000Hz (30.7%), and 6000Hz (29.5%). As expected for CI candidates, higher frequencies were associated with higher audiometric thresholds (Fig 4 and S2 Table).

### Quantity and distribution of sparsity

The amount of missing data significantly affects imputation accuracy. However, there is no standard for the amount of missing data that can be safely imputed. To address this, we first defined a clinically significant threshold for “safe” audiometric imputation (< 10dB RMSE). Model performance was then assessed with increasing amounts of missing data to quantify the amount of missing data that can be safely imputed. Another important consideration is the distribution of missing data. One common approach for comparing imputation algorithms is to take a MCAR assumption and randomly introduce missing values [28]. However, this does not reflect real-world audiometric data, as certain frequencies are significantly more commonly tested. To assess real-world performance, we tested randomly generated sparse datasets with sparsity distributions weighted to match that of our parent dataset. Three other sparsity distributions (random, terminal, central) were tested to assess potential generalizability to novel datasets with different sparsity distributions (Fig 3).

**Fig 5:**
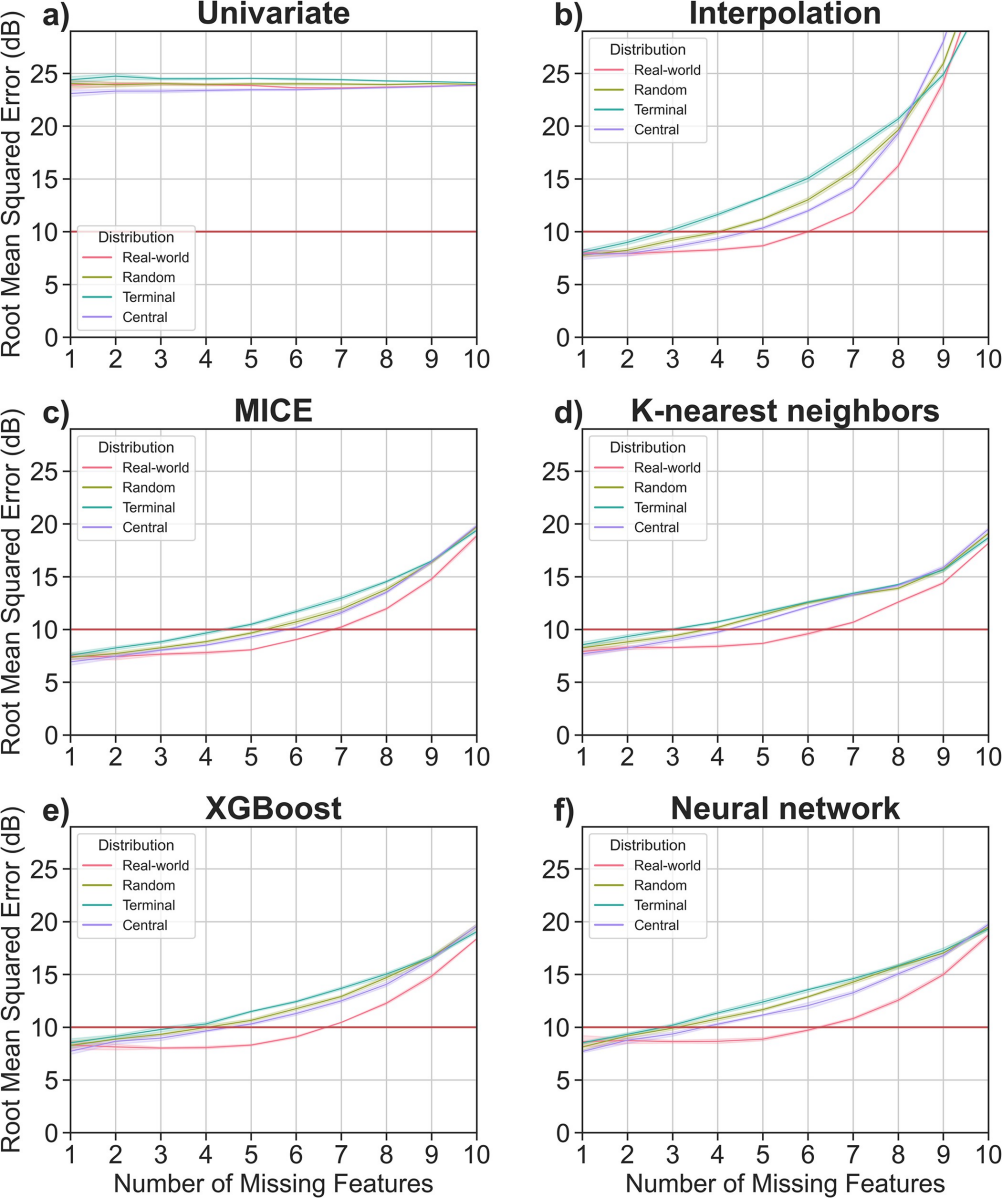
Quantity and distribution of sparsity. Assessment of model performance on sparse datasets with different degrees of sparsity (1–10 of 11 features) and sparsity distributions (Real-world, Random, Terminal, Central). Colored lines denote mean root mean squared error; shaded bands represent 99% confidence intervals.

As expected, model performance degraded with increasing amounts of missing data (Fig 5). With 3 to 8 missing features, all models (except UI) performed significantly better with a real-world sparsity distribution compared to random and weighted-random sparsity distributions (Δ RMSE 0.3 dB– 5.8 dB, non-overlapping 99% confidence intervals). With a real-world sparsity distribution, it is possible to safely impute up to 6 missing datapoints per audiogram using MICE, KNN, XGB, and NN. For all other sparsity distributions, the maximum for safe imputation ranged from 2 to 5 missing features, depending on model (excluding UI) and distribution (Fig 5). This highlights the importance of considering sparsity distribution when evaluating imputation models, particularly with audiometric data.

**Fig 6:**
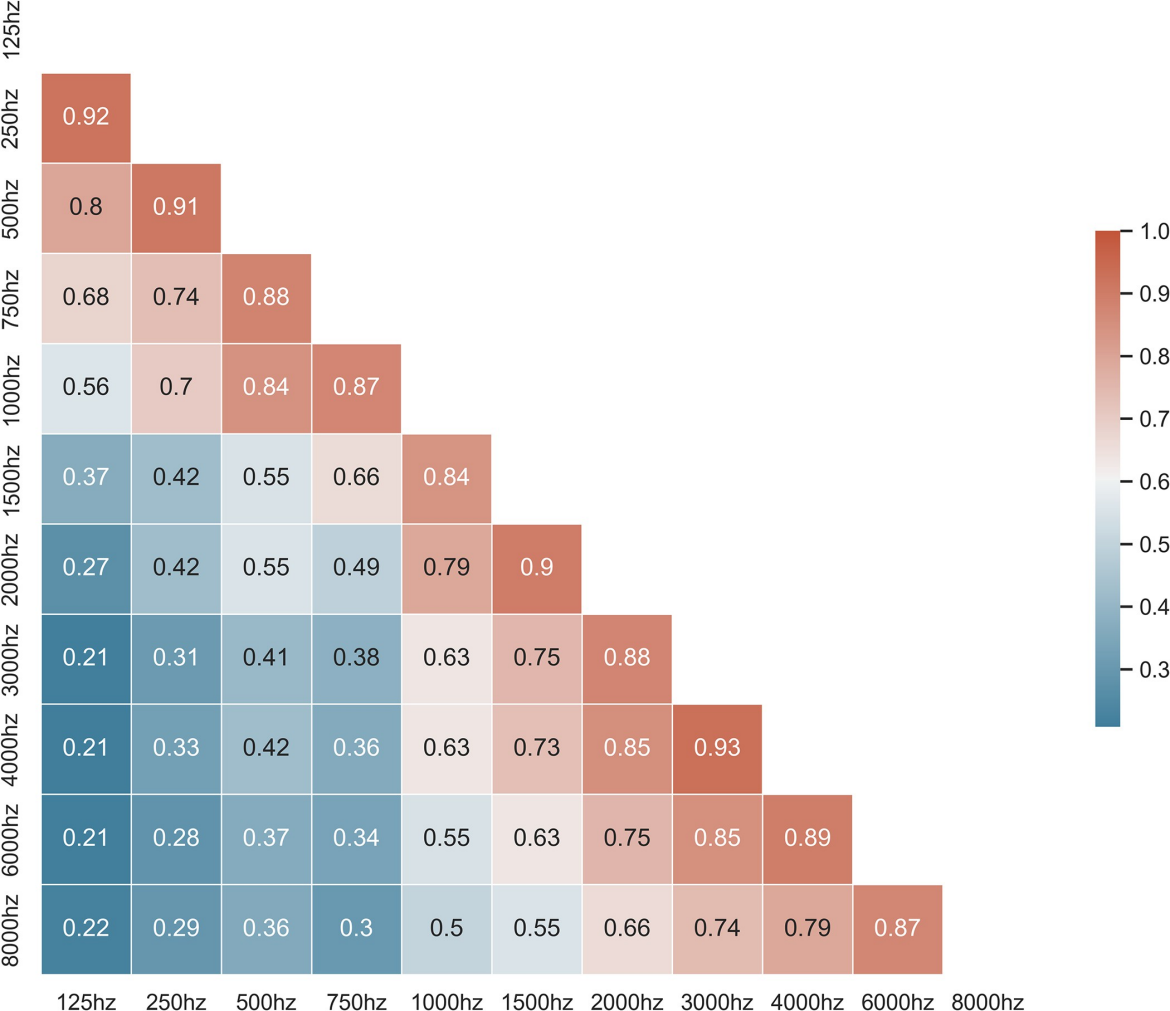
Audiometric correlation. Correlation matrix demonstrating the pairwise Pearson correlation coefficient between audiometric frequencies.

Audiometric data is highly multicollinear. Adjacent frequencies are most strongly correlated, and correlation strength falls off dramatically with increasing distance on the frequency spectrum (Fig 6). For example, the Pearson correlation coefficient between 125 Hz and 250 Hz is 0.92; comparatively, the correlation between 125 Hz and 3000 to 8000 Hz ranges from 0.21 to 0.22. We hypothesize that this is likely a driving factor behind the favorability of imputing missing audiometric data with a real-world sparsity distribution. Random and weighted-random sparsity distributions are statistically more likely to have multiple sequential missing frequencies, compared to the real-world distribution of sparsity which is strongly weighted towards alternating present (octave) and absent (inter-octave) frequencies (Figs 3 and 4D).

### Model selection

Sparsity of real-world data varies between instances both spatially (Fig 4D) and quantitatively (Fig 4A). To identify the best-performing model in a real-world scenario, models were tested on randomly generated sparse datasets with real-world spatial and quantitative distributions of sparsity, such that both the number and specific distribution of missing features varied between audiograms. Quantitative sparsity was capped at six missing features per instance, as this represents the maximum for safe imputation given a real-world spatial distribution of sparsity (Fig 5). MICE was the best performing model across all error metrics (p < 0.01, Wilcoxon rank sum test) with RMSE of 7.83 dB [95% CI 7.81–7.86] (Table 1). To assess generalizability of model selection to novel datasets, testing was repeated on randomly generated sparse datasets with other sparsity distributions (random, terminal, and central). As above, the number of missing features per instance was randomized on a per audiogram basis, capped at six but otherwise mirroring the quantitative sparsity of the parent subset. MICE was the best performing model across all error metrics (p < 0.01, Wilcoxon rank sum test) for each sparsity distribution tested (Table 1).

**Table 1:** Model selection. Model performance assessment given different sparsity distributions. Quantity of missing data varied on a per-instance basis, capped at 6 missing features but otherwise statistically equivalent to parent subset. Results averaged across 10 simulations, reported as mean (95% confidence interval).

Sparsity Distribution	Model	Mean Absolute Error	Root mean square error	R^2^ Score
Real-world	UI	18.9 (18.87–18.94)	23.97 (23.92–24.02)	-0.532 (-0.54 - -0.525)
	INT	5.22 (5.21–5.24)	8.34 (8.3–8.37)	0.918 (0.917–0.918)
	**MICE**	**5.08 (5.07–5.08)**	**7.83 (7.81–7.86)**	**0.93 (0.93–0.931)**
	KNN	5.38 (5.37–5.39)	8.34 (8.3–8.39)	0.92 (0.919–0.921)
	XGB	5.3 (5.29–5.31)	8.19 (8.15–8.23)	0.923 (0.922–0.923)
	NN	5.72 (5.7–5.74)	8.87 (8.82–8.92)	0.911 (0.91–0.912)
Random	UI	19.0 (18.98–19.02)	23.9 (23.87–23.92)	-0.458 (-0.463 - -0.452)
	INT	6.2 (6.19–6.22)	9.81 (9.76–9.87)	0.882 (0.881–0.883)
	**MICE**	**5.79 (5.77–5.81)**	**8.75 (8.72–8.79)**	**0.913 (0.912–0.914)**
	KNN	6.52 (6.5–6.54)	9.54 (9.51–9.58)	0.891 (0.89–0.892)
	XGB	6.75 (6.73–6.77)	9.87 (9.82–9.91)	0.885 (0.884–0.886)
	NN	7.73 (7.7–7.77)	11.34 (11.26–11.42)	0.855 (0.853–0.857)
Central	UI	18.53 (18.5–18.55)	23.2 (23.18–23.23)	-0.935 (-0.942 - -0.927)
	INT	5.97 (5.95–6.0)	9.16 (9.11–9.21)	0.881 (0.879–0.882)
	**MICE**	**5.6 (5.58–5.63)**	**8.39 (8.35–8.43)**	**0.906 (0.905–0.906)**
	KNN	6.47 (6.45–6.49)	9.33 (9.29–9.37)	0.877 (0.876–0.878)
	XGB	6.77 (6.74–6.8)	9.76 (9.71–9.8)	0.865 (0.864–0.866)
	NN	7.36 (7.34–7.39)	10.6 (10.56–10.65)	0.85 (0.848–0.852)
Terminal	UI	19.41 (19.38–19.44)	24.47 (24.43–24.51)	-0.217 (-0.219 - -0.214)
	INT	7.01 (6.97–7.04)	11.34 (11.26–11.41)	0.855 (0.854–0.857)
	**MICE**	**6.17 (6.14–6.21)**	**9.34 (9.29–9.39)**	**0.912 (0.911–0.913)**
	KNN	6.93 (6.9–6.95)	10.08 (10.03–10.12)	0.894 (0.893–0.895)
	XGB	7.13 (7.11–7.15)	10.48 (10.43–10.52)	0.887 (0.885–0.888)
	NN	7.89 (7.85–7.93)	11.57 (11.51–11.64)	0.866 (0.864–0.867)

**Bolded** values indicate best performing model for a given metric and spatial sparsity distribution (p < 0.01, Wilcoxon rank sum test). Models: Univariate (UI), interpolation (INT), multiple imputation by chained equations (MICE), k-nearest neighbors (KNN), gradient boosted trees (XGB), neural network (NN).

Prior literature states that machine learning models such as KNN and neural networks are optimal. These results directly conflict with this notion, indicating that simple iterative linear regression produces optimal results, and that complex machine learning methods are not just unnecessary but detrimental. Overall, audiometric data is highly multicollinear, making it an excellent candidate for imputation. This is further bolstered by the favorable sparsity distribution of real-world audiometric data. Imputing missing data in audiograms with up to 6 missing datapoints captures 99.3% of the audiograms in our dataset, allowing a 5.7-fold increase in sample size (1,304 to 7,399 audiograms) while maintaining a clinically insignificant level of error. These results also suggest an avenue for increased clinical efficiency for audiologists, as an accurate full 11-frequency audiogram could hypothetically be obtained by measuring only 5 frequencies and imputing the rest.

### Dataset size

**Fig 7:**
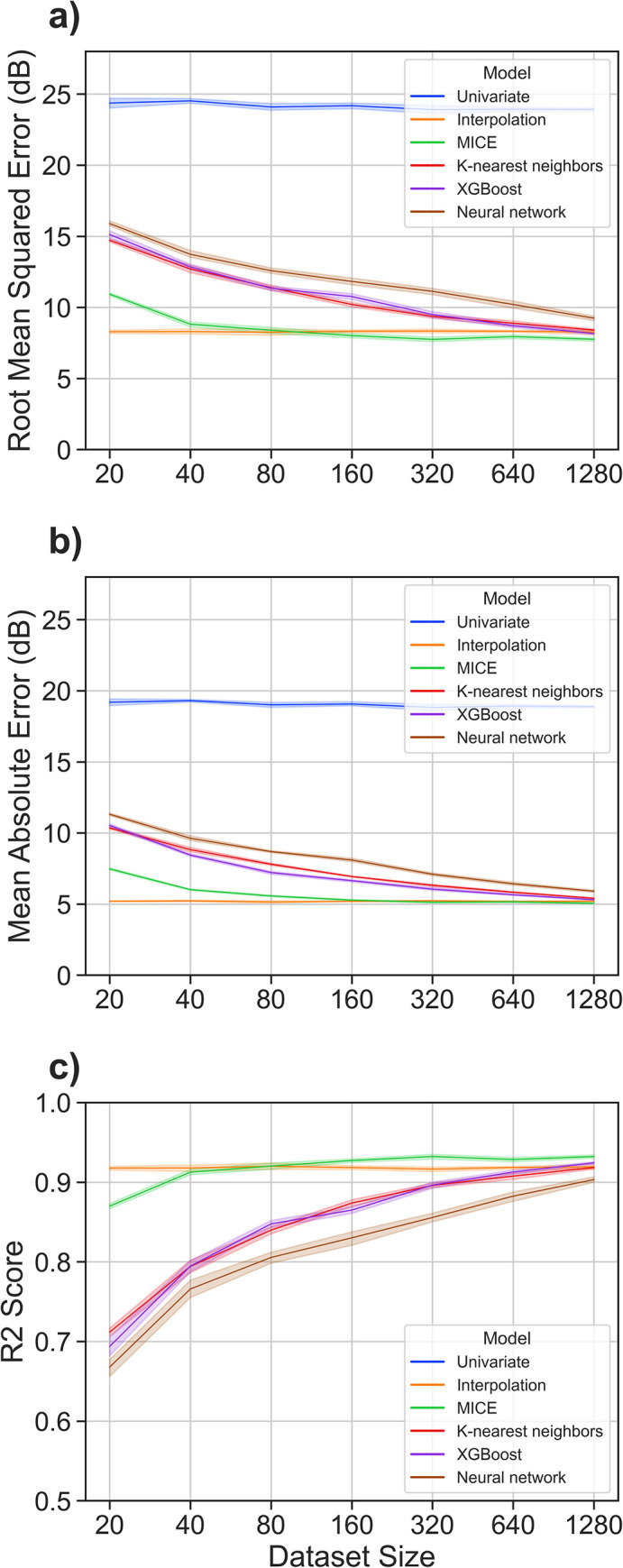
Model performance with varying dataset size. Models assessed on sparse datasets with sample size sequentially increasing twofold. Amount of sparsity varied on a per-instance basis from 1 to 6 missing features, mirroring the quantity of sparsity of the parent subset. Distribution of sparsity was real-world, mirroring parent subsets. Lines represent metric mean across 10 simulations; shaded bands represent 99% confidence intervals.

Dataset size is another consideration, as certain methods (e.g., neural networks) are known to require more data for optimal performance. Models were tested on datasets ranging in size from 20 to 1280 audiograms (Fig 7). Sparsity was capped at 6 missing datapoints per audiogram; otherwise, sparsity quantity and distribution were equivalent to the parent dataset. As expected, performance of UI and INT models was independent of dataset size; all other models displayed a positive correlation between dataset size and performance. For datasets smaller than ~1e2, INT was the best performing model. In all other cases, MICE was the best performing model. Notably, MICE performance plateaus at a dataset size of approximately 300–400 audiograms. In contrast, the more sophisticated machine learning models (KNN, XGB, and NN) continue to improve with increasing dataset size. This indicates the possibility that, with a significantly larger dataset, another model could outperform MICE. However, the performance of MICE on the entire dataset with up to 6 missing features is MAE 5.08 [95% CI 5.07–5.08] (Table 1). It is not possible to impute with greater accuracy than the stochastic noise (test-retest error) of the underlying dataset, which prior studies define as roughly +/- 5dB [17,18].

As such, MICE is close to the theoretical upper bound for model performance. For improvement with a more complex model to be significant enough to justify the cost of interpretability and ease of implementation, the literature-defined test-retest error would need to be an overestimate. However, a better model could potentially safely impute audiograms with greater than 6 missing datapoints. While this represents only 0.7% of audiograms in our dataset, this could be applicable for audiometric screening, potentially increasing audiologists’ clinical efficiency by decreasing the number of frequencies needed for a full audiogram.

### Limitations and future directions

Generalizability is a primary concern for implementation of any predictive model. While our dataset is well-represented, future datasets may have different structural sparsity as practices for audiometric testing change with time. As such, blind implementation is not recommended. An analysis of sparsity and model performance is critical to avoid inadvertently introducing bias. The attached GitHub repository contains code and instructions such that researchers may easily perform these analyses on novel datasets.

Audiometric testing practices also differ between institutions. Complete case analysis was used prior to analysis, potentially introducing selection bias. As institution-specific data is unavailable, the degree of selection bias could not be assessed. Furthermore, the study population was relatively homogenous; most patients were white or of unknown race with hearing loss predominantly of unknown etiology, all undergoing evaluation for cochlear implant. While results support imputation of other audiometric data, future work is needed to validate use in other populations. We hypothesize the decreased variance of audiometric thresholds in healthy patients would lead to increased performance.

Results were derived from imputation of full 11-frequency audiograms. However, investigators may decide to exclude certain frequencies. To assess generalizability of results with a restricted set of features, models were assessed for imputation of audiograms with 750 Hz and 1500 Hz (the two least commonly tested features) removed. This additionally allowed for assessment with a larger dataset, expanding sample size to 3,074. Results show that imputation with this feature set is comparatively more difficult. Given a real-world sparsity distribution, six non-missing datapoints were required for safe imputation, up from five non-missing datapoints with the 11-frequency dataset (S2 Fig). This suggests that a safer approach to the 11-frequency dataset would be capping quantitative sparsity at 5 rather than 6 missing features per instance, which still captures the majority (98.8%) of audiograms. Some may also disagree with a clinically significant threshold of ≥10dB RMSE. However, should investigators choose alternative definitions for clinically significant error, the threshold for error tolerance and the amount of missing data imputed may easily be adjusted.

Finally, models were optimized by grid-search. Certain models, such as XGB and NN, have many hyperparameters. A complete grid-search is not feasible, as grid-search size increases exponentially with additional parameters. As such, grid-search potentially underestimates performance of more complex models. To address this, we systematically identified all tunable hyperparameters prior to each test, such that 1) variance significantly (>1dB RMSE) affected model performance, and 2) there was no unilaterally superior value to serve as a default (S1 Table). Despite limitations, given the size of our dataset we believe the gain in generalizability from testing on all datapoints is worthwhile. Furthermore, our priority is avoiding performance overestimation, as a conservative understanding of performance is a key for incorporation of models into clinical research. Correspondingly, the parametric simplicity of INT, MICE, and KNN models represents an advantage over the more complex XGB and NN models. However, future work is needed to refine the process of identifying tunable hyperparameters, address the computational cost associated with grid search, and assess this approach to hyperparameter tuning across a range of datasets. Similarly, while the importance of sparsity distribution is evident in audiometric data, further work is needed to assess the effects of sparsity distribution on other datasets.

This study validates imputation of a significant amount of CI audiometric data, addressing the prevalent issue of missing data in CI outcomes research. A next step is implementation of imputation models to increase sample size for CI outcomes modeling. Finally, future work should assess the degree to which audiograms can be used to safely impute other preoperative factors known to be associated with audiometry, such as hearing loss duration and etiology, age, and speech perception measurements.

## Conclusions

Precision medicine has immense potential; however, these approaches are data-dependent and widespread sparsity in medical data represents a significant barrier to implementation. The current standard of complete case analysis leads to selection bias and decreased statistical power. The high degree of multicollinearity and favorable sparsity distribution of audiometric data makes it an excellent candidate for imputation. However, validation of imputation models is both necessary and often overlooked. We rigorously assessed the performance of six imputation models on CI candidate audiograms, demonstrating the importance of considering both the quantity and distribution of missing data. With a real-world sparsity distribution, up to 6 of 11 frequencies per audiogram can be safely imputed while maintaining RMSE below a clinically significant threshold of 10dB, allowing for a 5.7-fold increase in dataset size. The standard approach of complete case analysis leads to discarding of significant amounts of usable data. Univariate imputation, a common alternative to complete case analysis, introduces a significant amount of bias, is outperformed by all other models tested, and should not be used. Depending on dataset size and sparsity, optimal performance can be obtained with either interpolation or iterative linear regression. More sophisticated machine learning techniques, demonstrated by prior literature to be optimal, are unnecessary and detrimental. Results, derived using the largest CI dataset to date, are likely generalizable. However, the importance of validating imputation model performance on novel datasets prior to implementation cannot be understated.

## Supporting information

S1 Fig Interpolation models. Visualization of interpolants computed using linear, PCHIP, and cubic spline interpolation methods for a sample audiogram with six non-missing datapoints. Interpolants bounded by range of underlying data (0dB to 120dB).
(TIF)

S2 Fig 9-frequencies, rate and distribution analysis. Assessment of model performance on sparse datasets with different degrees of sparsity (1–8 of 9 features) and sparsity distributions (Real-world, Random, Terminal, Central). Colored lines denote mean root mean squared error; shaded bands represent 99% confidence intervals.
(TIF)

S1 Table Hyperparameter search space. Prior to each round of testing, tunable hyperparameters were identified, defined as hyperparameters for which no unilaterally superior default value exists and variance significantly (>1dB RMSE) affects performance. Tunable hyperparameters were identified for each test independently. This table is inclusive, reporting the intersection of all hyperparameter search spaces identified.
(DOCX)

S2 Table Demographics, numerical. Summary statistics for age, duration of hearing loss, audiometry, and speech perception testing. Measures of speech perception included consonant-vowel nucleus consonant (CNC) test and the Arizona Biomedical Sentence (AzBio) test in quiet and with +5dB or +10dB of background noise.
(DOCX)

## References

[1] NIDCD. NIDCD: Cochlear Implants 2021 [updated 3/24/21. Available from: https://www.nidcd.nih.gov/health/cochlear-implants.

[2] Boisvert I, Reis M, Au A, Cowan R, Dowell RC. Cochlear implantation outcomes in adults: A scoping review. PLoS One. 2020;15(5):e0232421–e. doi: 10.1371/journal.pone.0232421 32369519 PMC7199932

[3] Velde HM, Rademaker MM, Damen J, Smit AL, Stegeman I. Prediction models for clinical outcome after cochlear implantation: a systematic review. J Clin Epidemiol. 2021;137:182–94. doi: 10.1016/j.jclinepi.2021.04.005 33892087

[4] Nijman S, Leeuwenberg AM, Beekers I, Verkouter I, Jacobs J, Bots ML, et al. Missing data is poorly handled and reported in prediction model studies using machine learning: a literature review. J Clin Epidemiol. 2022;142:218–29. doi: 10.1016/j.jclinepi.2021.11.023 34798287

[5] Donders AR, van der Heijden GJ, Stijnen T, Moons KG. Review: a gentle introduction to imputation of missing values. J Clin Epidemiol. 2006;59(10):1087–91. doi: 10.1016/j.jclinepi.2006.01.014 16980149

[6] Hasan MK, Alam MA, Roy S, Dutta A, Jawad MT, Das S. Missing value imputation affects the performance of machine learning: A review and analysis of the literature (2010–2021). Informatics in Medicine Unlocked. 2021;27:100799.

[7] Ibrahim JG, Chu H, Chen MH. Missing data in clinical studies: issues and methods. J Clin Oncol. 2012;30(26):3297–303. doi: 10.1200/JCO.2011.38.7589 22649133 PMC3948388

[8] Little RJARDB. Statistical analysis with missing data. 2020.

[9] Greenland S, Finkle WD. A Critical Look at Methods for Handling Missing Covariates in Epidemiologic Regression Analyses. American Journal of Epidemiology. 1995;142(12):1255–64. doi: 10.1093/oxfordjournals.aje.a117592 7503045

[10] Roderick JAL. Regression With Missing X’s: A Review. Journal of the American Statistical Association. 1992;87(420):1227–37.

[11] Charih F, Steeves A, Bromwich M, Mark AE, Lefrançois R, Green JR, editors. Applications of Machine Learning Methods in Retrospective Studies on Hearing. 2018 IEEE Life Sciences Conference (LSC); 2018 28–30 Oct. 2018.

[12] Pitathawatchai P, Chaichulee S, Kirtsreesakul V. Robust machine learning method for imputing missing values in audiograms collected in children. Int J Audiol. 2022;61(1):66–77. doi: 10.1080/14992027.2021.1884909 33641573

[13] Schafer EC, Grisel JJ, de Jong A, Ravelo K, Lam A, Burke M, et al. Creating a framework for data sharing in cochlear implant research. Cochlear Implants Int. 2016;17(6):283–92. doi: 10.1080/14670100.2016.1253246 27882827

[14] Kohavi R. A Study of Cross-Validation and Bootstrap for Accuracy Estimation and Model Selection. 2001;14.

[15] Bradford JP, Brodley CE. The Effect of Instance-Space Partition on Significance. Machine Learning. 2001;42(3):269–86.

[16] Cawley G, Talbot N. On Over-fitting in Model Selection and Subsequent Selection Bias in Performance Evaluation. Journal of Machine Learning Research. 2010;11:2079–107.

[17] Schlauch RS, Carney E. A multinomial model for identifying significant pure-tone threshold shifts. J Speech Lang Hear Res. 2007;50(6):1391–403. doi: 10.1044/1092-4388(2007/097) 18055764

[18] Schmuziger N, Probst R, Smurzynski J. Test-retest reliability of pure-tone thresholds from 0.5 to 16 kHz using Sennheiser HDA 200 and Etymotic Research ER-2 earphones. Ear Hear. 2004;25(2):127–32. doi: 10.1097/01.aud.0000120361.87401.c8 15064657

[19] Konrad-Martin D, James KE, Gordon JS, Reavis KM, Phillips DS, Bratt GW, et al. Evaluation of audiometric threshold shift criteria for ototoxicity monitoring. J Am Acad Audiol. 2010;21(5):301–14; quiz 57. doi: 10.3766/jaaa.21.5.3 20569665 PMC5588921

[20] Virtanen P, Gommers R, Oliphant TE, Haberland M, Reddy T, Cournapeau D, et al. SciPy 1.0: fundamental algorithms for scientific computing in Python. Nature Methods. 2020;17(3):261–72. doi: 10.1038/s41592-019-0686-2 32015543 PMC7056644

[21] Pedregosa F, Varoquaux G, Gramfort A, Michel V, Michel V, Grisel O, et al. Scikit-learn: Machine Learning in Python. Journal of Machine Learning Research. 2011;12:28225–2830.

[22] Chen TaG Carlos. Proceedings of the 22nd ACM SIGKDD International Conference on Knowledge Discovery and Data Mining. XGBoost: A Scalable Tree Boosting System 2016 [Available from: http://doi.acm.org/10.1145/2939672.2939785.

[23] Kotsiantis SB, Zaharakis ID, Pintelas PE. Machine learning: a review of classification and combining techniques. Artificial Intelligence Review. 2006;26(3):159–90.

[24] Pedregosa F, Varoquaux G, Gramfort A, Michel V, Thirion B, Grisel O, et al. Scikit-learn: Machine Learning in Python. J Mach Learn Res. 2011;12(null):2825–30.

[25] Parthasarathy A, Romero Pinto S, Lewis RM, Goedicke W, Polley DB. Data-driven segmentation of audiometric phenotypes across a large clinical cohort. Scientific Reports. 2020;10(1):6704. doi: 10.1038/s41598-020-63515-5 32317648 PMC7174357

[26] Jakobsen JC, Gluud C, Wetterslev J, Winkel P. When and how should multiple imputation be used for handling missing data in randomised clinical trials–a practical guide with flowcharts. BMC Medical Research Methodology. 2017;17(1):162. doi: 10.1186/s12874-017-0442-1 29207961 PMC5717805

[27] Khan SI, Hoque ASML. SICE: an improved missing data imputation technique. Journal of Big Data. 2020;7(1):37. 32547903 10.1186/s40537-020-00313-w PMC7291187

[28] Schmitt P, Mandel J, Guedj M. A comparison of six methods for missing data imputation. Journal of biometrics & biostatistics. 2015;6(1).

