# Supplementary figures and images for: Imputation of missing values for cochlear implant candidate audiometric data and potential applications
Source: PLoS One. 2023 Feb 6;18(2):e0281337. doi: 10.1371/journal.pone.0281337 (PMC9901781; doi:10.1371/journal.pone.0281337)

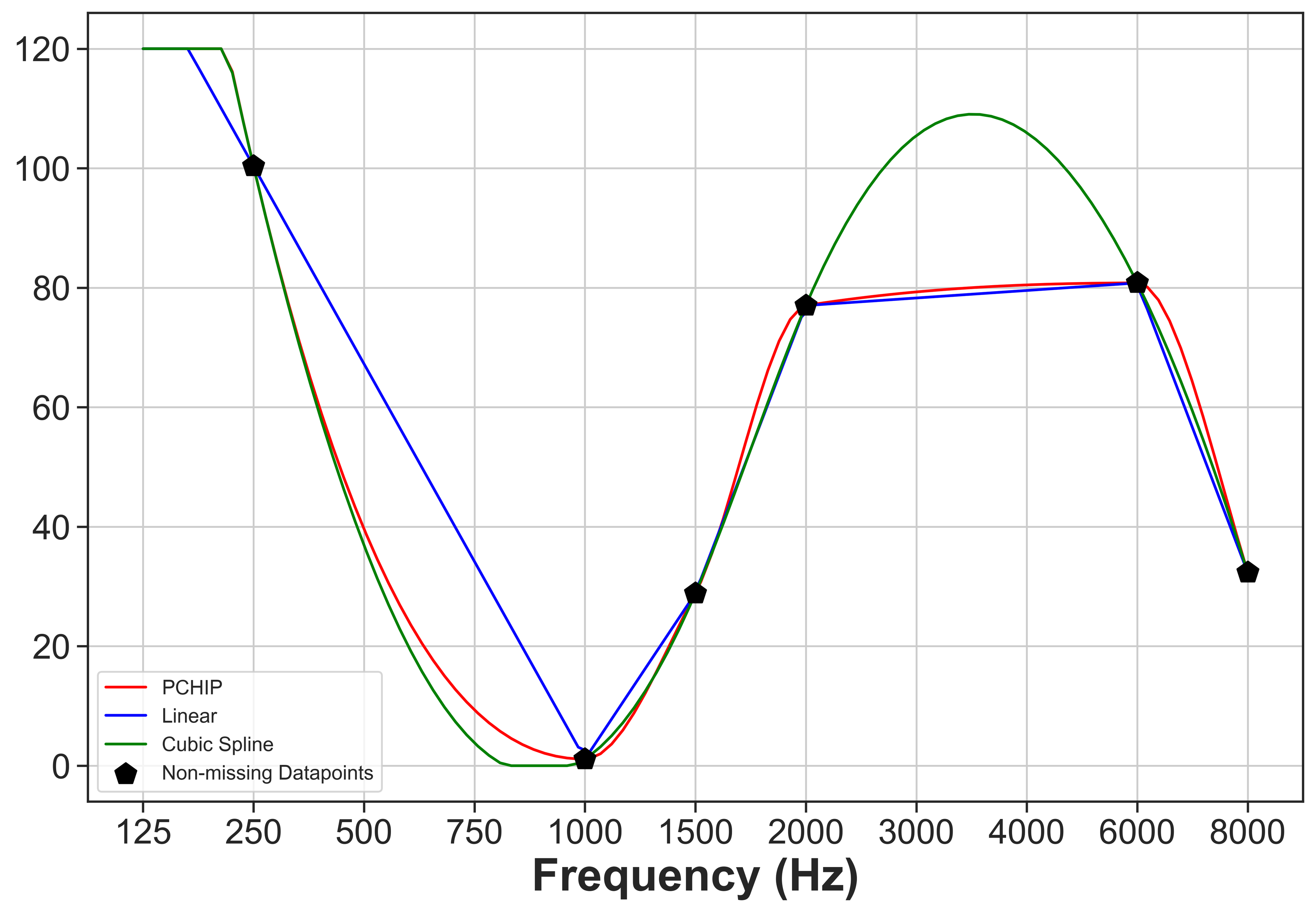

Supplement: S1 Fig — Visualization of interpolants computed using linear, PCHIP, and cubic spline interpolation methods for a sample audiogram with six non-missing datapoints. Interpolants bounded by range of underlying data (0dB to 120dB). (TIF) [file pone.0281337.s001.tif]

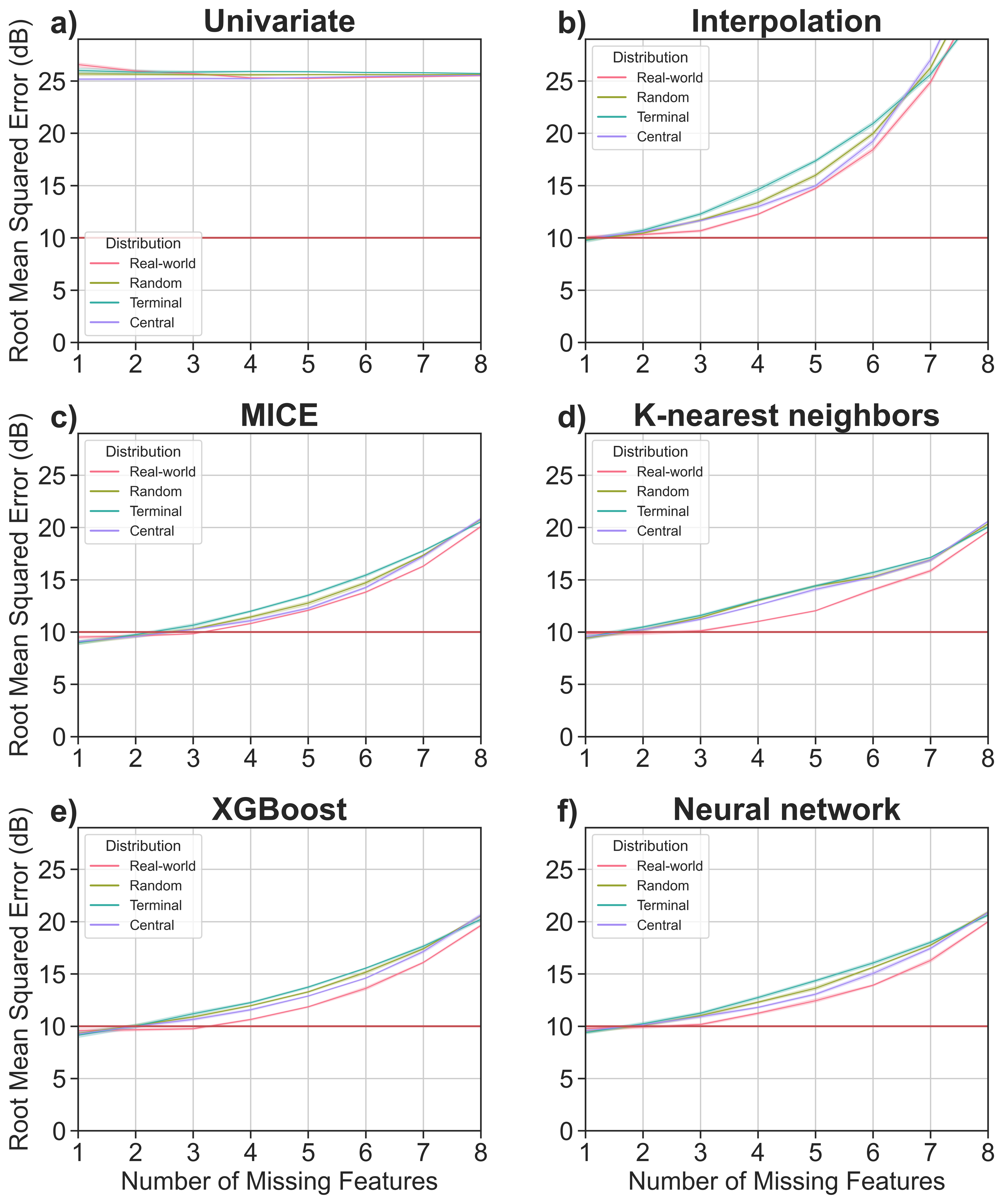

Supplement: S2 Fig — Assessment of model performance on sparse datasets with different degrees of sparsity (1–8 of 9 features) and sparsity distributions (Real-world, Random, Terminal, Central). Colored lines denote mean root mean squared error; shaded bands represent 99% confidence intervals. (TIF) [file pone.0281337.s002.tif]
