# Supplementary material for: Imputation of missing values for cochlear implant candidate audiometric data and potential applications
Source: PLoS One. 2023 Feb 6;18(2):e0281337. doi: 10.1371/journal.pone.0281337 (PMC9901781; doi:10.1371/journal.pone.0281337)
Supplement: S1 Table — Prior to each round of testing, tunable hyperparameters were identified, defined as hyperparameters for which no unilaterally superior default value exists and variance significantly (>1dB RMSE) affects performance. Tunable hyperparameters were identified for each test independently. This table is inclusive, reporting the intersection of all hyperparameter search spaces identified. (DOCX) [file pone.0281337.s003.docx]

| **Model** | **Hyperparameter** | **Search Space** | **Description** |
| --- | --- | --- | --- |
| Univariate | Strategy | Mean  Median  Mode | Univariate method by which missing values are imputed. ‘Mean’, ‘Median’, and ‘Mode’ impute missing values within a feature as equivalent to the feature mean, median, or mode, respectively. |
| Interpolation | Method | Linear  PCHIP  Cubic Spline | If ‘Linear’, interpolants are linear functions. If ‘PCHIP’ (piecewise cubic hermite interpolating polynomial) or ‘Cubic Spline’, interpolants are piecewise cubic polynomials with continuous first (‘PCHIP’, ‘Cubic Spline’) and second (‘Cubic Spline’ only) derivatives. |
| MICE | Estimator | Lasso  Ridge | Multivariate estimator applied iteratively to predict missing values. If ‘Lasso’, estimator is a l1-weighted linear regressor. If ‘Ridge’, estimator is a l2-weighted linear regression model. Strength of regularization (λ) is set by an additional nested round of cross-validation. |
|  | Iterations (*m*) | 3 – 15 | Number of imputation rounds performed. |
|  | Initial imputation | Linear  Mean | Single imputation function for initial round of imputation. Necessary as multivariate estimators are intolerant of missing values. ‘Linear’ indicates linear interpolation, ‘Mean’ indicates feature mean. |
|  | Output | Final  SICE | Method by which MICE model output is derived. ‘Final’ indicates that the *m*th round of imputation is taken as output. ‘SICE’ indicates that the output is defined as the average of all *m* imputation rounds. |
| KNN | N neighbors (*k*) | 5 – 100 | Number of nearest neighbors identified for each instance. |
|  | Weight (*w*) | Distance  Uniform | Function used to weight contribution of nearest neighbors. If ‘Uniform’, neighbors are weighted equally. If ‘Distance’, neighbors are weighted proportionally to the inverse of their distance in Euclidean space from the imputed instance. |
| XGBoost | N estimators | 400 – 900 | Number of gradient boosted decision trees created. Higher values tend to produce more accurate models, with diminishing returns. |
|  | Max depth | 3 – 6 | Maximum decision tree depth. Higher values create more complex models that are potentially more accurate but more likely to overfit. |
|  | Learning rate | 0.005 – 0.015 | Shrinkage term applied to feature weights after each boosting round. |
|  | Subsample | 0.25 – 0.75 | Randomly sampled proportion of data seen by each decision tree. |
| Neural Network | Layers | 2 - 4 | Number of hidden layers in the neural network. |
|  | Nodes | 32 - 256 | Number of neurons in each hidden layer |
|  | Learning rate | 0.005 - 0.015 | Initial learning rate; determines step size when updating weights. |

**S1 Table: Hyperparameter search space**. Prior to each round of testing, tunable hyperparameters were identified, defined as hyperparameters for which no unilaterally superior default value exists and variance significantly (>1dB RMSE) affects performance. Tunable hyperparameters were identified for each test independently. This table is inclusive, reporting the intersection of all hyperparameter search spaces identified.
