# Supplementary material for: Imputation of missing values for cochlear implant candidate audiometric data and potential applications
Source: PLoS One. 2023 Feb 6;18(2):e0281337. doi: 10.1371/journal.pone.0281337 (PMC9901781; doi:10.1371/journal.pone.0281337)
Supplement: S2 Table — Summary statistics for age, duration of hearing loss, audiometry, and speech perception testing. Measures of speech perception included consonant-vowel nucleus consonant (CNC) test and the Arizona Biomedical Sentence (AzBio) test in quiet and with +5dB or +10dB of background noise. (DOCX) [file pone.0281337.s004.docx]

|  | **Count** | **Mean** | **Std** | **Min** | **25%** | **Median** | **75%** | **Max** |
| --- | --- | --- | --- | --- | --- | --- | --- | --- |
| **Age** | 7451.0 | 64.2 | 16.7 | 18.0 | 54.0 | 68.0 | 77.0 | 96.0 |
| **Hearing loss duration** | 6795.0 | 24.6 | 17.9 | 0.0 | 10.0 | 21.0 | 36.0 | 89.0 |
| **125hz** | 4100.0 | 57.2 | 30.7 | 0.0 | 35.0 | 50.0 | 75.0 | 120.0 |
| **250hz** | 7399.0 | 63.3 | 29.5 | 0.0 | 40.0 | 60.0 | 85.0 | 120.0 |
| **500hz** | 7406.0 | 70.4 | 28.2 | 0.0 | 50.0 | 70.0 | 90.0 | 120.0 |
| **750hz** | 2566.0 | 74.9 | 24.4 | 0.0 | 60.0 | 75.0 | 95.0 | 120.0 |
| **1000hz** | 7401.0 | 80.8 | 25.8 | 0.0 | 65.0 | 80.0 | 100.0 | 120.0 |
| **1500hz** | 2009.0 | 89.2 | 22.6 | 0.0 | 75.0 | 90.0 | 110.0 | 120.0 |
| **2000hz** | 7400.0 | 90.6 | 25.7 | 0.0 | 75.0 | 95.0 | 115.0 | 120.0 |
| **3000hz** | 5128.0 | 94.6 | 24.3 | 0.0 | 75.0 | 100.0 | 120.0 | 120.0 |
| **4000hz** | 7386.0 | 97.7 | 24.2 | 0.0 | 80.0 | 105.0 | 120.0 | 120.0 |
| **6000hz** | 5213.0 | 101.1 | 23.8 | 0.0 | 85.0 | 110.0 | 120.0 | 120.0 |
| **8000hz** | 6955.0 | 104.4 | 23.6 | 0.0 | 90.0 | 120.0 | 120.0 | 120.0 |
| **CNC** | 4422.0 | 23.1 | 23.4 | 0.0 | 2.0 | 16.0 | 36.0 | 100.0 |
| **AzBio Quiet** | 4049.0 | 29.2 | 30.1 | 0.0 | 0.0 | 20.0 | 49.0 | 100.0 |
| **AzBio +5dB** | 655.0 | 20.6 | 22.1 | 0.0 | 3.0 | 14.0 | 29.0 | 98.0 |
| **AzBio +10dB** | 1902.0 | 24.0 | 26.3 | 0.0 | 0.0 | 16.0 | 36.0 | 100.0 |

**S2 Table. Demographics, numerical.** Summary statistics for age, duration of hearing loss, audiometry, and speech perception testing. Measures of speech perception included consonant-vowel nucleus consonant (CNC) test and the Arizona Biomedical Sentence (AzBio) test in quiet and with +5dB or +10dB of background noise.
